# Oxidative Stress in Autistic Children Alters Erythrocyte Shape in the Absence of Quantitative Protein Alterations and of Loss of Membrane Phospholipid Asymmetry

**DOI:** 10.1155/2018/6430601

**Published:** 2018-11-11

**Authors:** Alessandra Bolotta, Michela Battistelli, Elisabetta Falcieri, Alessandro Ghezzo, Maria Cristina Manara, Stefano Manfredini, Marina Marini, Annio Posar, Paola Visconti, Provvidenza Maria Abruzzo

**Affiliations:** ^1^Department of Experimental, Diagnostic and Specialty Medicine, Bologna University, Via Belmeloro 8, 40126 Bologna, Italy; ^2^IRCCS Fondazione Don Carlo Gnocchi, Via A. Capecelatro 66, 20148 Milan, Italy; ^3^Department of Biomolecular Sciences, Urbino University, Via A. Saffi 2, 61029 Urbino, Italy; ^4^CRS Development of Biomolecular Therapies, Experimental Oncology Laboratory, Istituto Ortopedico Rizzoli, Via di Barbiano 1/10, 40136 Bologna, Italy; ^5^Department of Life Sciences and Biotechnologies, Ferrara University, Via G. Savonarola 9, 44121 Ferrara, Italy; ^6^Department of Biomedical and Neuromotor Sciences, Bologna University, Via U. Foscolo 7, 40123 Bologna, Italy; ^7^Child Neurology and Psychiatry Unit, IRCCS Istituto delle Scienze Neurologiche di Bologna, Via Altura 3, 40139 Bologna, Italy

## Abstract

Red blood cells (RBCs) from people affected by autism spectrum disorders (ASDs) are a target of oxidative stress. By scanning electron microscopy, we analyzed RBC morphology from 22 ASD children and show here that only 47.5 ± 3.33% of RBC displayed the typical biconcave shape, as opposed to 87.5 ± 1.3% (mean ± SD) of RBC from 21 sex- and age-matched healthy typically developing (TD) controls. Codocytes and star-shaped cells accounted for about 30% of all abnormally shaped ASD erythrocytes. RBC shape alterations were independent of the anticoagulant used (Na_2_-EDTA or heparin) and of different handling procedures preceding glutaraldehyde fixation, thus suggesting that they were not artefactual. Incubation for 24 h in the presence of antioxidants restored normal morphology in most erythrocytes from ASD patients. By Coomassie staining, as well as Western blotting analysis of relevant proteins playing a key role in the membrane-cytoskeleton organization, we were unable to find differences in RBC ghost composition between ASD and normal subjects. Phosphatidylserine (PS) exposure towards the extracellular membrane domain was examined in both basal and erythroptosis-inducing conditions. No differences were found between ASD and TD samples except when the aminophospholipid translocase was blocked by N-ethylmaleimide, upon which an increased amount of PS was found to face the outer membrane in RBC from ASD. These complex data are discussed in the light of the current understanding of the mode by which oxidative stress might affect erythrocyte shape in ASD and in other pathological conditions.

## 1. Introduction

The erythrocyte plasma membrane has unique properties, which allow the cell to provide an extended surface for gaseous exchanges and to undergo large passive deformations while the erythrocyte squeezes itself through narrow capillaries, some of them with cross sections one-third its own diameter. These unusual properties are due to the complexity of the structural network supporting the plasma membrane, where the phospholipid bilayer is anchored to a two-dimensional spectrin hexagonal lattice via protein junctional complexes centered on band 3, the anion-exchange channel. Two major complexes connect band 3 with the cytoskeletal spectrin network, the ankyrin complex and the actin complex, but, according to a recent review [1], the composition of these band 3-associated protein complexes is not constant. On the overall, the red cell membrane contains about 20 major proteins and at least 850 minor ones [1]. A recent paper [2] pointed out the role of nonmuscle myosin IIA in maintaining erythrocyte shape by interacting with the actin network associated with band 3 complexes.

The membrane structure, which assures both shape resiliency and a marked physiological deformability, also allows RBC to undergo unique and reversible shape changes, from discocytes to spherical globes (spherocytes), or to concave (stomatocytes), or to crenated (echinocytes) shapes. These changes are triggered by a variety of chemical and physical agents (including pH and ATP concentration) and, in certain conditions, can even occur cyclically in sequence [3]. In his paper, Rudenko [3] extensively discusses RBC shape transitions, pointing out that two main theories have been advanced to explain them: (i) one based on the bilayer couple of biological membranes, which suggests that any effect that expands the outer leaflet relative to the inner one produces a tendency to form convex structures on the cell surface (e.g., echinocytic spicules), whereas an expansion of the inner leaflet relative to the outer one favors concavities (e.g., stomatocytic shapes) [4, 5]; (ii) the other based on changes in band 3 conformation, leading to altered ionic composition within the cell [6, 7]. However, recent research [8] challenged the current theories linking in a straightforward way RBC shape alterations to disturbances of the membrane-cytoskeleton network.

A number of pathological conditions are associated with characteristic RBC shape alterations, which, at variance with Rudenko's transitions, tend to be stable over time [9]. For instance, typical thorny red cells (acanthocytes) are prevalent in neuroacanthocytosis, a group of rare genetic diseases [10]; hereditary spherocytosis, elliptocytosis, and stomatocytosis are RBC disorders resulting from mutations in genes encoding various membrane and skeletal proteins [11]; codocytes are a common occurrence in beta-thalassemia [12], which is also characterized by oxidative stress [13]. Leptocytes and other abnormal erythrocyte shapes were found in Rett patients [14], a genetic neurodevelopmental disorder accompanied by oxidative stress and hypoxia. A marked beta-actin deficiency was afterwards described in RBC from these patients [15]. The same team also described the presence of abnormal RBC shapes and, in a less convincing way, of decreased beta-actin expression in “classical” (i.e., nonsyndromic) autistic patients [16]. “Classical” autism is the most common of the neurodevelopmental disorders characterized by social and behavioral impairments and collectively named autism spectrum disorders (ASDs). ASD has a prevalent genetic etiology; however, epigenetically acting environmental factors (e.g., immune dysregulation, pollutants) seem to have a key role in the development of the disease [17]. Oxidative stress is a distinctive feature of ASD [18, 19] and deeply affects RBC membrane, impairing its fluidity, altering its lipid composition, and affecting the activity on Na^+^/K^+^-ATPase [18, 20].

In patients with chronic obstructive pulmonary disease, chronic oxidative stress alters RBC shape, but normal shape is reestablished following appropriate antioxidant medication [21]. Similarly, RBC storage in blood banks leads to the build-up of reactive oxygen species (ROS) and of oxidation products such as malondialdehyde. The ensuing oxidative stress leads to the formation of spiculated cells (echinocytes), to the budding of micro- and nanovesicles from the spiculae [22], and eventually to hemolysis; all of which may be prevented by supplementation with antioxidants [23]. Moreover, oxidative stress triggers PS externalization [24], but with modalities still not clarified.

Understanding how oxidative stress alters RBC morphology—and possibly function—requires the evaluation of how it affects the band 3-centered cytoskeletal network. It has been shown that oxidative stress, as well as other stimuli, increases phosphorylation of band 3 on tyrosine residues, thus markedly reducing band 3 affinity for ankyrin, leading to the disruption of the membrane skeletal architecture and favoring progressive plasma membrane vesiculation [25]. However, in the presence of oxidative stress, the antioxidant enzyme peroxiredoxin-2 (Prx2) progressively migrates to the RBC plasma membrane, where it associates with the amino domain of band 3 [26, 27]. The band 3 amino domain is also the regulatory site where glucose metabolism is addressed towards the glycolytic ATP production or, conversely, towards the production of molecules offering antioxidant protection—NADPH and GSH [28]. Thus, PRX2 binding to the band 3 sites allows at the same time ATP production and its own antioxidant protection to the membrane site.

This work is aimed at understanding the extent of RBC shape alteration in ASD, its molecular base(s), and how it may affect RBC functions. As we recently demonstrated [20] that the amount of beta-actin in RBC from ASD children did not differ from that of TD controls, we turned our attention to the quantification of band 3 and stomatin. These proteins were chosen because band 3 has a pivotal role in cytoskeletal organization, while stomatin was among the few RBC membrane proteins found to be differentially expressed in ASD leukocytes [29].

## 2. Materials and Methods

### 2.1. Subjects

Twenty-two children diagnosed with nonsyndromic ASD (17 males and 5 females, age (mean ± SD) 7.75 ± 1.87 years, age range 5.25–11.08 years) and 21 typically developing children (14 males and 7 females, age (mean ± SD) 9.44 ± 1.96 years, age range 5.25–11.83 years) were recruited by the Child Neuropsychiatry Unit of the Bellaria Hospital (IRCCS, Bologna) within a larger study approved by Local Ethical Committee (Azienda USL Bologna, Imola, Ferrara, CE 13062, 23/07/2013; Prot. N.1198/CE). The study was conducted according to the Declaration of Helsinki guidelines. Written consent was obtained from parents as well as from children through pictures and simplified information. The patients were affected by nonsyndromic autism, according to a complete clinical diagnostic assessment (ADOS, DSM-5) and a comprehensive neurological workup, detailed in a previous manuscript by our group [30]. Moreover, patients were subjected to a complete pediatric assessment, including the evaluation of hematological parameters and of electrolytes, which ruled out the presence of iron deficiency (occasionally reported to occur in ASD children [31]), hemolysis, and splenomegaly. Control typically developing (TD) children were recruited in the same local community and were free of cognitive, learning, and psychiatric problems. Subjects did not take any dietary supplement or medication in the 4 months preceding the evaluation and were on a typical Mediterranean diet. Demographic and clinical data are shown in Tables 1(a) and 1(b).

### 2.2. Materials

Unless otherwise specified, chemicals were analytical grade and purchased from Sigma-Aldrich, St. Louis, MO.

### 2.3. RBC Preparation and Morphological Evaluation

From each subject, two blood samples (1 mL) were collected, one in Na_2_-EDTA and the other in sodium heparin vacutainers. For morphological evaluation with scanning electron microscopy, we always treated the samples within 1 h from collection. We compared two preparation protocols. In the first protocol, aimed at limiting mechanical stress to the cells, 30 *μ*L of whole blood was transferred to an Eppendorf 1.5 mL tube, centrifuged (5 min at 100 ×g) in order to separate plasma, which was gently removed with a micropipette. Cells were fixed in suspension with 2.5% glutaraldehyde in cold 0.1 M phosphate buffer pH 7.2 for 2 h at room temperature. During this step, RBC underwent spontaneous sedimentation. The supernatant was then gently removed with a micropipette, phosphate buffer was added, and the sample was maintained at 4°C till successive steps. In the “rougher” protocol, 30 *μ*L of RBC suspension was obtained after Ficoll density gradient and three washing steps in phosphate-buffered saline. The RBC suspension was then fixed in 2.5% glutaraldehyde in 0.1 M phosphate buffer pH 7.2 as described for the more gentle protocol. Cells were quickly washed in 0.15 M phosphate buffer at pH 7.2, and then drops of the suspension were deposited on poly-L-lysine-coated coverslips [32]. The adhesion was carried out overnight in a moist and sealed chamber at 4°C. Afterwards, the slides were washed and postfixed with 1% OsO_4_ in the same buffer for 1 h. A gentle progressive alcohol dehydration was performed, and specimens were critical point-dried. After mounting on conventional SEM stubs by means of silver glue, specimens were gold sputtered, by a sputtering device [33] and finally observed with a Philips 515 scanning electron microscopy. The morphological alterations were evaluated by two different observers, in separate observation sessions. The entire slide was evaluated, and then twenty microscope fields, for each sample, were photographed at the same magnification (1770x magnitude). The images were used for the classification of erythrocytes according to their morphology.

### 2.4. Antioxidant In Vitro Treatment

This study was carried out in a subgroup of subjects, 6 ASD and 8 TD. RBC suspensions obtained from Ficoll gradient and washed three times in phosphate-buffered saline were used. RBC suspensions were diluted in 2 mL SAGM storage medium (NaCl 8.77 g/L; adenine 0.169 g/L; dextrose 9 g/L; mannitol 5.25 g/L) at the concentration of 250 *μ*L RBC/mL. Three parallel samples, 2.5 mL each, were seeded in 6-well culture plates (9.6 cm^2^/well surface area). The RBC suspension was (i) treated with an antioxidant mix containing 8 *μ*M (final dilution) tocotrienol (Carlo Sessa SpA, Milan, Italy) dissolved at 1000x concentration in dimethyl sulphoxide (DMSO) and 0.7 *μ*M (final dilution) Q10 (ACEF SpA, Fiorenzuola D'Arda, Italy) dissolved 1000x in N,N-dimethylformamide (DMF); (ii) untreated; and (iii) additioned with DMSO plus DMF alone. The cell suspensions were incubated 24 h at 37°C in 5% CO_2_ atmosphere. 40 *μ*L was then collected from each well, centrifuged (5 min at 100 ×g) to remove the SAGM solution, and fixed in 2.5% glutaraldehyde solution for scanning electron microscopy as described above.

*Ghost suspension* was prepared as previously described [20].

*SDS-polyacrylamide gel electrophoresis* (SDS-PAGE) was carried out with ghost suspensions; gels were loaded semiquantitatively (equal volume of packed ghosts/lane), as described [34]. Precast gradient gels (Mini-PROTEAN TGX Stain-Free Protein Gel, 4–15% polyacrylamide, Bio-Rad Laboratories, Hercules, CA) were used. Some gels were stained with Coomassie Brilliant Blue-R250 (Bio-Rad, Hercules, CA).

*Western blotting* was performed following SDS-PAGE and transferred to nitrocellulose membranes [20]. The following primary antibodies were used: mouse monoclonal anti-beta-actin (C4) (Santa Cruz Biotechnology, Dallas, Texas) at 1 : 1000 dilution; mouse monoclonal anti-stomatin (E-5) (Santa Cruz Biotechnology, Dallas, Texas) at 1 : 1000 dilution; and mouse monoclonal SLC4A1, antianion exchanger 1 or band 3 (IVF12) (DSHB Hybridoma Product IVF12, deposited to the DSHB by Jennings M.L. at 1 : 30000 dilution). Secondary antibody was ImmunoPure® Goat Anti-Mouse IgG, (H + L), peroxidase conjugated (Thermo Scientific, Waltham, Massachusetts), and was used at the following dilutions: 1 : 5000, 1 : 15000, and 1 : 20000, respectively. ECL: Western Bright™ ECL (Advansta, Menlo Park, CA) was used for detection. Specific protein band density was quantified by means of Bio-Rad GelDoc 2000 with reference to the actin band.

### 2.5. Evaluation of Phosphatidylserine Exposure

This study was carried out in a subgroup of subjects, 11 ASD and 10 TD. Phosphatidylserine (PS) exposure was evaluated by flow cytometry. The procedure outlined by Kuypers et al. [35] was followed, with small modifications. RBCs from a Ficoll gradient suspension were initially diluted with Hanks' balanced salt solution (HBSS) and distributed into 6 tubes which were examined in the following conditions: (1) basal, untreated; (2) N-ethylmaleimide (NEM); (3) osmotic shock (OS); (4) OS + NEM; (5) Ca^2+^ ionophore A23187; and (6) ionophore A23187 + NEM. For samples (2), (4), and (6), RBCs (30% hematocrit) were incubated with 10 mM NEM for 30 min at 37°. After incubation, the samples were washed in HBSS and then diluted at 16% hematocrit before the evaluation or the successive treatments. OS was performed at 16% hematocrit 10 min at room temperature with 9 g/100 mL NaCl. For the treatment with Ca^2+^ ionophore 23187, RBCs at 16% hematocrit were previously loaded with 1 mM CaCl_2_ for 3 min in incubator, and then the ionophore was added at 4 *μ*M final concentration; the samples were incubated at 37°C for 20 min, and then the reaction was stopped with 2.5 mM Na_2_-EDTA. Cells were then washed 3 times with HBSS buffer +1% BSA. Before loading the samples to the cytometer, the samples were diluted in 10 mL phosphate-buffered saline, and then 1 mL was centrifuged (5 min at 400 ×g) and the pellet was incubated with FITC-labelled annexin V using the annexin V-FLUOS Staining Kit (Roche, Basel, Switzerland) according to the manufacturer's instructions. Samples were acquired using a FACSCalibur instrument and analyzed with the CellQuest Software (Becton Dickinson, Italy).

### 2.6. Statistical Analysis

Data were tested for normality using GraphPad Prism7, which employed the D'Agostino-Pearson test, following which appropriate parametric tests (Student's *t* for independent data) or the nonparametric equivalent (Mann-Whitney) was used. Differences were considered significant at *p* < 0.05.

## 3. Results

### 3.1. Scanning Electron Microscopy Reveals Altered Erythrocyte Shape in RBC from ASD Subjects

**Figure 1:**
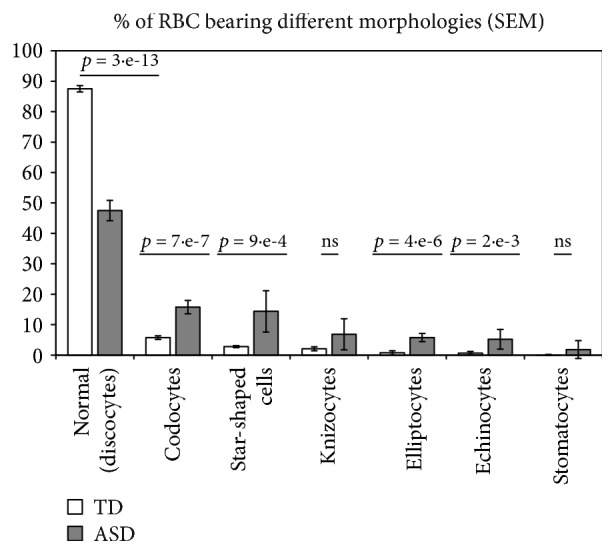
RBC bearing different morphologies, as percent of total RBC. Open columns: RBC from typically developing children. Dark columns: RBC from children affected by autistic spectrum disorder. Mean ± SD. Numbers over the columns indicate whether the difference was statistically significant (*p* < 0.05 by Student *t*-test).

**Figure 2:**
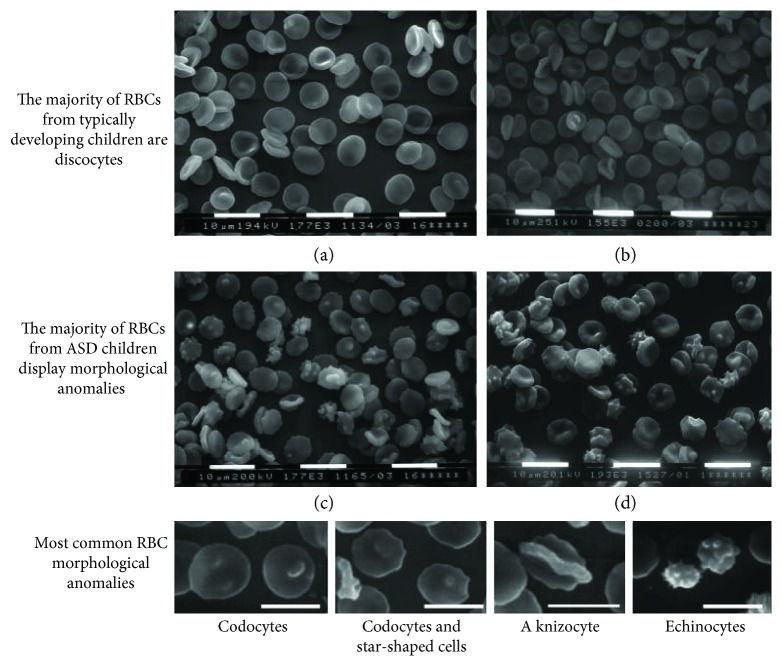
RBC morphology at scanning electron microscopy. (a, b) Normal RBC morphology (discocytes) found in typically developing children. (c, d) A variety of RBC abnormal morphologies found in children affected by autistic spectrum disorder. Bottom panel: a gallery of abnormal RBC morphologies.

Figure 1 shows the distribution of RBC by morphology in TD and in ASD children. Most RBC (87.5 ± 1.3%, mean ± SD) from TD children displayed the typical normal biconcave shape, which characterized only 47.5 ± 3.33% (mean ± SD) of RBC from ASD children. The difference was highly significant (*p* = 3∗*e* ^−13^). The extremely low variability among the samples makes impossible to correlate morphological shapes to clinical features, which display a much higher variability. Among abnormally shaped RBC, codocytes and star-shaped cells were the most represented ones, whereas echinocytes were relatively few. Abnormal morphologies were not peculiar of RBC from ASD subjects, rather ASD subjects displayed abnormal RBC shapes in a higher percentage of erythrocytes with respect to healthy subjects. Representative images of microscopic fields form one TD and from one ASD subject, as well as a gallery of abnormal morphologies are shown in Figure 2.

Notably, no difference was found with respect to the anticoagulant used or the prefixation procedures.

### 3.2. Incubation with Antioxidants Restores Normal Morphology to RBC from ASD Subjects

**Figure 3:**
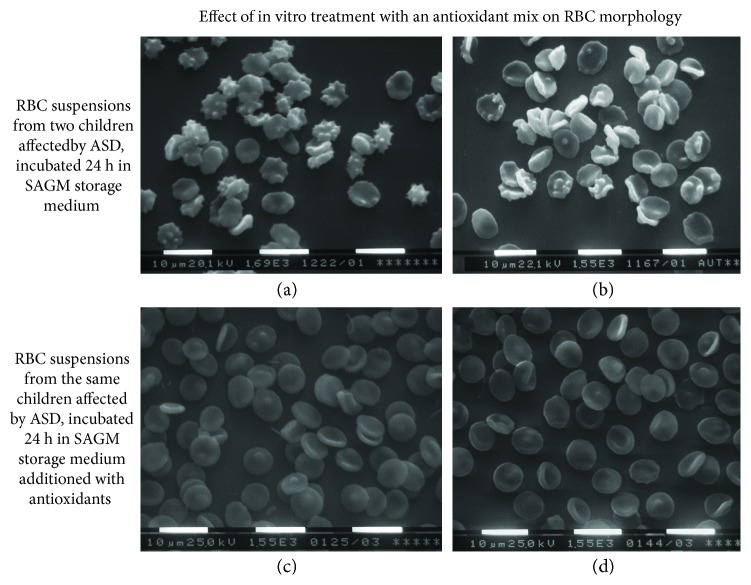
Effect of antioxidant treatment on the morphology of RBC from children affected by autistic spectrum disorder. (a, b) Before the in vitro 24 h treatment. (c, d) After the treatment with tocotrienol and Q10. The percentage of abnormally shaped erythrocytes is clearly decreased.

In vitro treatment with the antioxidant mix brought the percentage of discocytes from 47.5 to 82%, thus substantially restoring the normal morphology to control levels. A representative image is shown in Figure 3. Incubation in SAGM alone or additioned with the DMSO + DMF vehicle did not alter the original RBC morphology.

### 3.3. Qualitative and Semiquantitative Evaluations of Ghost Proteins Do Not Show Appreciable Differences between ASD and TD Subjects

**Figure 4:**
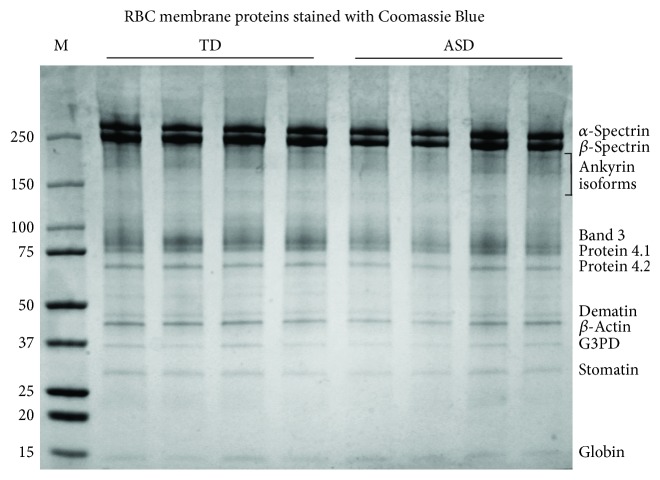
A representative SDS-PAGE stained with Coomassie Blue. The gel was a precast 4–15% polyacrylamide gel. M: markers; TD: RBC samples from typically developing children; ASD: RBC samples from children affected by autistic spectrum disorder. On the left side: MW of the markers. On the right side: bands presumably corresponding to major RBC proteins.

Ghost extracts were run in SDS-polyacrylamide gradient gels and stained with Coomassie Blue in order to allow an overview of the protein composition of the extracts. As shown in Figure 4, no difference could be appreciated at a qualitative level. In particular, bands 4.1 and 4.2, which were reported to be differentially expressed in ASD leukocytes [29], display the same staining intensity in TD and ASD samples.

**Figure 5:**
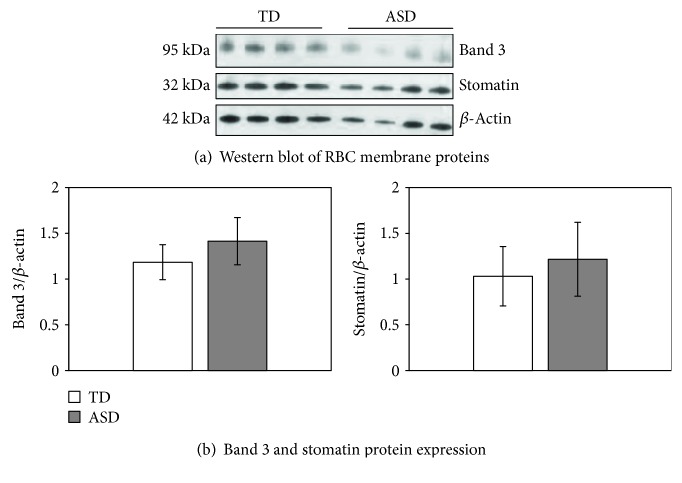
(a) A representative Western blot where anti-band 3 and anti-stomatin antibodies were used to quantitate specific proteins separated by SDS-PAGE; *β*-actin was used as loading control. The gel was a precast 4–15% polyacrylamide gel. TD: four RBC samples from typically developing children; ASD: four RBC samples from children affected by autistic spectrum disorder. (b) Densitometry showing the intensity of specific protein bands relative to the corresponding *β*-actin band. Ghost samples from all patients and controls were examined. Differences between TD and ASD RBC were not statistically significant.

We recently reported that TD- and ASD-derived RBC did not vary in beta-actin amount, as evaluated by Western blotting performed according to the most rigorous standards [20]. This result allowed us to use beta-actin as reference protein to quantitate other proteins by Western blotting. We chose to evaluate band 3 and stomatin, basing the choice on the motivations mentioned in Introduction.  Figure 5 reports representative WB results for the two proteins. Band intensity of the two examined proteins, as quantified relative to beta-actin, was the same in TD- and ASD-derived ghost samples.

### 3.4. Phosphatidylserine Exposure Does Not Seem to Differ in Erythrocytes between TD and ASD Children, but Addition of N-Ethylmaleimide Suggests That ASD RBC Undergo an Increased PS Flip-Flop Cycling in Basal Conditions

**Figure 6:**
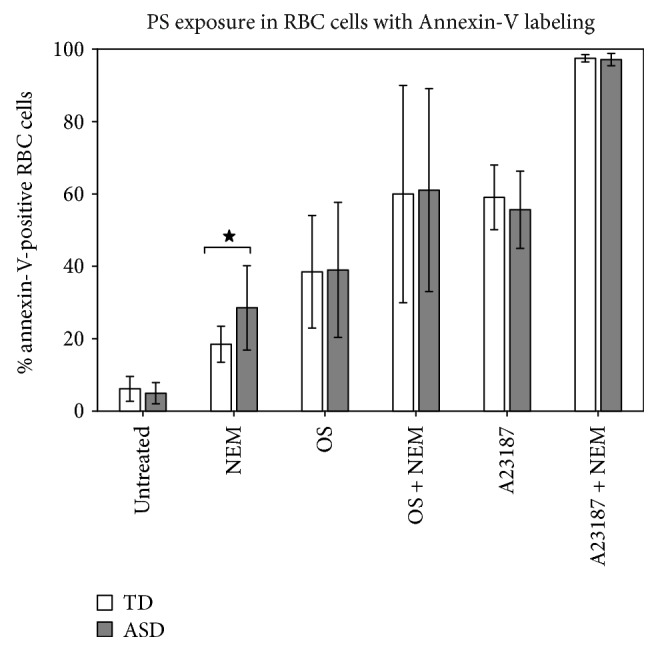
Percent of annexin V (+) RBC in typically developing children (TD, open columns) or in children affected by autistic spectrum disorder (ASD, dark columns). Treatments are shown under the columns: NEM: N-ethylmaleimide; OS: osmotic shock; A23187: calcium ionophore. Mean ± SD. Star: difference was statistically significant (*p* = 0.03 by Student *t*-test).

The localization of PS on the outer side of the lipid bilayer was assessed in order to evaluate the tendency of RBC from ASD children to undergo erythroptosis, as a consequence of oxidative stress and as a result of morphological anomalies, both in basal conditions (i.e., without any treatment) and when exposed to either osmotic stress or calcium overload. Results, reported in Figure 6, show that no difference in PS exposure is present in basal conditions. However, when the aminophospholipid translocase was blocked by NEM, a significant (*p* = 0.03) increase in PS exposure could be appreciated in ASD RBC, suggesting that in basal conditions, PS flippase was actively engaged in reimporting PS from the outer bilayer. The addition of NEM to osmotic stressed or to calcium overloaded RBC was not able to unveil any difference between RBCs from TD and ASD subjects, although one should remark the rather high variability in percent PS exposure following OS and the maximal PS exposure as a response to calcium overload, both situations where a ≈50% increase of PS exposure could have gone unnoticed.

## 4. Discussion

In the present work, we addressed the question of how oxidative stress, which affects ASD subjects altering in multiple ways RBC and their plasma membrane [18–20, 30], impinges on RBC shape, composition of the plasma membrane-cytoskeletal network and propensity to undergo erythroptosis. Although the group of Ciccoli et al. had reported multiple shape anomalies and a decrease in *β*-actin in RBC from “classical autistic” subjects [16], our experience with hyperspectral microscopy [30] was not confirming the presence of shape alterations at a large extent; moreover, our accurate evaluation of *β*-actin content had not revealed differences between RBCs from TD and ASD children [20]. By studying the extensive literature on the determinants of erythrocyte shape alterations, we understood that the apparent lack of morphological alterations found with hyperspectral microscopy was probably to be ascribed to the so-called “glass effect,” as described by Wong [6] that likely cancels the difference between TD and ASD RBC morphology. In fact, when RBC shape changes are “fixed” by abnormal aggregation or clusterization of RBC proteins, as in beta-thalassemia or in sickle cell anemia, the “glass effect” does not take place; on the other hand, the contact with the glass slide in isosmotic unbuffered medium favors ionic concentration adjustments and makes very hard to distinguish the difference between TD and ASD RBC morphology in both fresh blood samples and standard Giemsa-stained slides. Thus, scanning electron microscopy is better suited for the morphological analysis of RBC shape alterations, as those described by the group of Ciccoli et al. By a careful study, which took into consideration some of the possible artifacts that could affect erythrocyte shape, and in particular by comparing with the same modalities ASD and TD RBC, we confirmed the findings of Ciccoli et al. group about shape anomalies in more than half of the RBC from autistic children. Remarkably, these abnormal morphologies were (i) independent of the anticoagulant used, confirming that only a long conservation in Na_2_-EDTA may affect RBC shape [36], and (ii) unaffected by repeated preparative centrifugations, carried out in buffered saline. Of note, RBC from ASD did not display bizarre or unique morphologies; rather they had a higher percentage of altered morphologies found in lesser amounts in RBC from TD subjects. In particular, one would expect to find a prevalence of echinocytes, since there are numerous reported instances where discocytes exposed to oxidative stress turned into echinocytes [25, 26]. This however was not the case with ASD RBC, although their exposure to ROS is indisputable. It is our opinion that ASD RBC do not assume the echinocyte shape because they *do not* loose ATP, at least at the extent lost by RBC in the abovementioned models of oxidative stress.

An eye evaluation of ghost proteins separated by SDS-PAGE and stained by Coomassie Blue does not suggest the occurrence of major differences in the protein set between TD and ASD subjects. This has been demonstrated by us for *β*-actin [20] and for band 3 and stomatin in the present work. However, in order to study the ≈870 different proteins located at the erythrocyte plasma membrane and to find possible expression differences between TD and ASD subjects, one should use an -omic approach; proteomics allowed Mohanty et al. [37] to find many differentially expressed proteins in RBC from Alzheimer patients and healthy individuals. On the other hand, using a transcriptomic approach, Glatt et al. [29] found a relevant number of differentially expressed genes in leukocytes from ASD patients. Nevertheless, the fact that incubation with an antioxidant mix was able to almost completely restore the normal morphology in ASD RBC points to oxidative stress-induced modifications of the protein-to-protein associations or of membrane proteins themselves rather than to major quantitative differences.

The most obvious culprit of the morphological anomalies would be band 3 protein, which, as discussed above, undergoes multiple phosphorylations in an oxidative environment, thus altering its interaction with the other proteins of the junctional complexes [25]. However, other proteins are likely to be involved as well; for instance, it may be worth investigating whether the newly found actin-myosin IIA interaction [2] is affected by oxidative stress. Advanced glycation end products are found in the plasma of ASD children [19], but the presence of glycated proteins in ASD erythrocytes has not been evaluated. For instance, spectrin is easily glycated [38] and its glycation would be a normal occurrence in steady-state conditions, due to the high-glucose concentration and the rich-oxygen milieu of the erythrocyte. Spectrin glycation occurs at sites of PS binding; thus, translocation of PS from the inner to the outer lipid monolayer not only provides the basis for erythroptosis but also contributes to alteration of the spectrin-based membrane skeleton, to shape modification and to decreased deformability of the cell. Such events do not occur in steady-state conditions since an ATP-powdered aminophospholipid translocase is continuously relocating PS to the inner membrane side [39]. In a murine model of *β*-thalassemia [13], oxidative stress leads to massive PS externalization and to erythrocyte loss. Indeed, our cytometric data strongly suggest not only an increased PS externalization in ASD RBC but also its efficient contrast by the activity of aminophospholipid translocases.

## 5. Conclusions

As far as we know so far, oxidative stress in ASD children is not generated within the erythrocyte and might be a consequence of chronic low-level (neuro)inflammation. Despite the extensive shape anomalies, the enhanced bilayer stiffness, the reduced activity of its Na^+^/K^+^-ATPase, and the increased needs to replenish the ATP supply required to maintain an accelerated activity of aminophospholipid translocases, it is amazing that oxidative stress at the erythrocyte level seems to be limited in consequences, as suggested by the fact that no clinical sign of reduced erythrocyte activity (e.g., impaired peripheral tissue oxygenation) has been so far described in ASD patients.

At a mere speculative level, we advance the hypothesis that a key role may be played by the interplay of PRX2 with the N-terminal domain of band 3. As oxidative stress promotes the migration of PRX2 to the membrane and its binding to the N-terminal domain of band 3, glucose metabolism is addressed towards the glycolytic ATP production for a longer fraction of time or in more numerous membrane locations, thus supplying the energy required for maintaining membrane phospholipid symmetry. Moreover, RBC antioxidant defenses are so abundant that RBCs are able to cope with many threats brought by exogenous ROS.

## Figures and Tables

**Table 1a:** (a) Demographic and clinical features of the ASD children

No.	Gender	Age (months)	Onset pattern: 1 (early); 2 (regressive); 3 (mixed)	Brief nonverbal IQ^∗^	ADOS score^∗∗^	CARS total score	CARS activity level item score	CARS body use (stereotypies) item score	CARS verbal communication item score	CARS nonverbal communication item score	CARS total number of items whose score was ≥3
1	M	68	1	48	20	41.5	2.5	3	3	3	11
2	M	94	1	52	21	46	3.5	4	4	4	11
3	F	68	3	71	20	43.5	2.5	3	3.5	3	10
4	M	93	2	45	20	48.5	3	4	3.5	3	12
5	M	82	2	55	19	42	3	3	3	3	9
6	M	115	1	52	19	39.5	3	3	3	3	7
7	M	74	3	68	19	41	2.5	3	3	3	9
8	M	99	1	56	16	35	2.5	2	3	3	3
9	M	85	1	62	22	41	2.5	2	3	3	7
10	F	74	1	47	17	38	2	2.5	3	3	7
11	F	124	3	87	21	33.5	2	2	2.5	2.5	4
12	M	129	1	68	19	39	2	2.5	3	3	7
13	M	78	3	82	17	39	3	2.5	3	3	6
14	M	96	1	67	22	44	2.5	3	3	3	11
15	M	84	1	95	18	39.5	2.5	3	3	3	8
16	M	114	1	45	21	47	3	3.5	3.5	3	13
17	M	103	3	47	16	41.5	4	2	3	3	10
18	M	131	1	96	18	35	2	3	2.5	2.5	3
19	F	64	1	75	17	39.5	2.5	2.5	3.0	3.0	6.0
20	F	64	1	74	17	41.0	2.5	2.5	3.0	3.0	7.0
21	M	98	1	49	20	41	2	3.5	2.5	2	9
22	M	133	3	80	13	34	2	1.5	3	2.5	2

^∗^Cognitive level: ≥70, normal; 50–69, mild cognitive impairment; 35–49, moderate cognitive impairment; and <35, severe cognitive impairment. (ICD-10 classification of mental and behavioural disorders, WHO, 1992). ^∗∗^ADOS modules 1 or 2 (total score autism cutoff = 12).

**Table 1b:** (b) Demographic features of the typically developing children

No.	Gender	Age (months)
1	F	140
2	F	74
3	F	97
4	F	93
5	M	142
6	M	127
7	M	116
8	M	142
9	M	135
10	M	63
11	F	125
12	M	115
13	M	115
14	M	125
15	F	102
16	F	120
17	M	101
18	M	130
19	M	123
20	M	136
21	M	65

## Data Availability

The data used to support the findings of this study are available from the corresponding author upon request.
